# Depletion of O6-alkylguanine-DNA alkyltransferase correlates with potentiation of temozolomide and CCNU toxicity in human tumour cells.

**DOI:** 10.1038/bjc.1993.241

**Published:** 1993-06

**Authors:** J. C. Baer, A. A. Freeman, E. S. Newlands, A. J. Watson, J. A. Rafferty, G. P. Margison

**Affiliations:** CRC Department of Medical Oncology, Charing Cross Hospital, London, UK.

## Abstract

Temozolomide (8-carbamoyl-3-methylimidazo[5,1-d]-1,2,3,5-tetrazin-4-(3H)-one) has shown promising activity in Phase I trials against some brain (glioma) and skin (melanoma, mycosis fungoides) cancers. Temozolomide and lomustine (CCNU) showed parallel toxicity in seven human tumour cell lines and this generally correlated (correlation coefficients 0.87 and 0.92 respectively) with the level of expression of the DNA repair protein O6-alkylguanine-DNA alkyltransferase (ATase, EC 2.1.1.63). Pretreating cells with the ATase inhibitor, O6-benzylguanine (BG), potentiated cytotoxicity to a similar degree with both drugs, but did not sensitise a cell line (ZR-75-1) expressing very low levels of this protein. When BG pretreatment was combined with repeat doses of temozolomide a dramatic potentiation (300 fold) was seen in MAWI cells, which express high levels of ATase, but not in a cell line (U373) expressing lower levels of ATase. [14C]-labelled temozolomide uptake was similar in sensitive and resistant lines. Human ATase-cDNA transfected xeroderma pigmentosum (XP) fibroblasts were more resistant than XP control cells to temozolomide and the related chloroethylating agent mitozolomide and although BG completely suppressed ATase activity in these cells, resistance was still greater than in control cells.


 © Macmillan Press Ltd., 1993

### Depletion of O$^6$-alkylguanine-DNA alkyltransferase correlates with potentiation of temozolomide and CCNU toxicity in human tumour cells

J.C. Baer[1], A.A. Freeman[1], E.S. Newlands[1], A.J. Watson[2], J.A. Rafferty[2] & G.P. Margison[2]

[1]*CRC Department of Medical Oncology, Charing Cross Hospital, Fulham Palace Road, London W6 8RF, UK and* [2]*CRC Department of Carcinogenesis, Paterson Institute for Cancer Research, Christie Hospital NHS Trust, Manchester M20 9BX, UK.*

**Summary** Temozolomide (8-carbamoyl-3-methylimidazo[5,1-d]-1,2,3,5-tetrazin-4-(3H)-one) has shown promising activity in Phase I trials against some brain (glioma) and skin (melanoma, mycosis fungoides) cancers. Temozolomide and lomustine (CCNU) showed parallel toxicity in seven human tumour cell lines and this generally correlated (correlation coefficients 0.87 and 0.92 respectively) with the level of expression of the DNA repair protein O$^6$-alkylguanine-DNA alkyltransferase (ATase, EC 2.1.1.63). Pretreating cells with the ATase inhibitor, O$^6$-benzylguanine (BG), potentiated cytotoxicity to a similar degree with both drugs, but did not sensitise a cell line (ZR-75-1) expressing very low levels of this protein. When BG pretreatment was combined with repeat doses of temozolomide a dramatic potentiation (300 fold) was seen in MAWI cells, which express high levels of ATase, but not in a cell line (U373) expressing lower levels of ATase. [$^{14}$C]-labelled temozolomide uptake was similar in sensitive and resistant lines. Human ATase-cDNA transfected *xeroderma pigmentosum* (XP) fibroblasts were more resistant than XP control cells to temozolomide and the related chloroethylating agent mitozolomide and although BG completely suppressed ATase activity in these cells, resistance was still greater than in control cells.

Temozolomide (CCRG 81045, NSC 362856) has recently completed an extended Phase I trial at Charing Cross Hospital, London and Queen Elizabeth Hospital, Birmingham (Newlands *et al.*, 1992). It was selected for clinical testing due to a combination of its broad spectrum activity against a range of murine tumours including P388 and L1210 leukaemias, M5076 sarcoma and B16 melanoma and its limited bone marrow toxicity (Stevens *et al.*, 1987). In the clinic temozolomide has shown activity against high grade glioma, malignant melanoma and mycosis fungoides. Of particular interest is its activity in a pilot study of patients with primary brain tumours, who had relapsed following radiotherapy (O'Reilly *et al.*, in press). The drug exhibits marked schedule dependency and has little activity when given as a single dose. The recommended dose is 750 - 1000 mg m$^{-2}$, given orally, split over 5 days and repeated over a 4 week cycle (Newlands *et al.*, 1992).

Temozolomide rapidly degrades in physiological solutions to form the reactive methylating species, MTIC (Stevens *et al.*, 1984) which reacts with DNA bases forming methyl addition products chiefly at N$^7$-guanine, N$^3$-adenine and O$^6$-guanine (Bull, 1988). O$^6$-alkylguanine is repaired by the protein O$^6$-alkylguanine DNA alkyltransferase (ATase) which captures the alkyl group onto one of its own cysteine residues in a stoichiometric autoinactivating reaction (Tano *et al.*, 1990). There is increasing evidence that O$^6$-alkylguanine is a major cytotoxic lesion following exposure to methylating and chloroethylating agents: for example, in ATase deficient cells, bacterial (Margison & O'Connor, 1990) or mammalian ATase cDNA transfection (Wu *et al.*, 1992) confers resistance to these agents. If, as would appear likely, temozolomide has a similar mechanism of cell killing, one possible method of potentiating its cytotoxicity would be to deplete the ATase protein. In the present study we have investigated the relationship between temozolomide cytotoxicity, ATase expression and the effect of O$^6$-benzylguanine (BG), an inhibitor of ATase (Dolan *et al.*, 1991).

### Materials and methods

### Materials

Tissue culture medium was purchased from ICN Biomedicals Ltd (High Wycombe, UK) and foetal calf serum from Gibco Ltd (Paisley, UK). O$^6$-benzylguanine (BG) was kindly supplied by Dr R.C. Moschel (NCI-Frederick Cancer Research & Development Center, Frederick, Maryland, USA). Temozolomide and its chloroethyl analogue, mitozolomide (8-carbamoyl-3-(2-chloroethyl) imidazo [5,1-d]-1,2,3,5-tetrazin-4-(3H)-one), were synthesised by May and Baker Ltd (Dagenham, UK) and stored as solutions in DMSO at − 70°C. All other chemicals were purchased from Sigma Chemical Co. Ltd. (Poole, UK).

### Cytotoxicity studies

Cell lines were routinely grown as monolayers in DMEM supplemented with 10% foetal calf serum, 25 mM HEPES, glutamine and penicillin/streptomycin. Cytotoxicity studies were carried out in HEPES-free medium in a 5% $CO_2$ atmosphere. 750-1000 cells/well were plated in 96 well plates and after overnight incubation were treated for 2 h with or without 33 μM BG. Temozolomide or CCNU was then added for 1 h in the same medium, the final DMSO concentration not exceeding 1%. The cells were grown for a further 7 days in fresh medium and assayed for protein content by the NCI sulphorhodamine assay (Skehan *et al.*, 1990; Wasserman & Twentyman, 1988); growth studies showed that cells were in log phase growth during the assay period. For the repeat temozolomide dosing schedule cells were given consecutive 24 h treatments, with fresh medium each day. Assays were carried out at least in duplicate.

Human ATase cDNA-transfected or control XP cells (Fan *et al.*, 1991) were grown in MEM and 1000 cells/well were plated. After a 3 h incubation, temozolomide, freshly diluted into MEM was added and the plates incubated for 5 days. Survivals were assayed as previously described (Morten *et al.*, 1992). In the BG experiments 300 cells were plated in triplicate onto 9 cm plates and allowed to attach for 5 h. BG was added (10 μM in MEM) 3 h prior to treatment with temozolomide which was freshly diluted into MEM containing 10 μM BG. After 7 days colonies were stained with Giemsa and counted.

### *O$^6$-alkylguanine DNA alkyltransferase assay*

This was carried out as described previously (Lee *et al.*, 1991). The basis of the assay is the incubation of cell extracts with DNA which contains O$^6$-methylguanine labelled with [$^3$H] in the methyl group after which the DNA is degraded to acid soluble material and the protein, which contains the methylated ATase, is collected by centrifugation and counted. The protein content of the cells was determined

Correspondence: E.S. Newlands.

with a BioRad protein assay kit using bovine serum albumin as a standard.

*Cellular uptake of [$^{14}$C]-labelled temozolomide*

8-carbamoyl-3-[$^{14}$C]methylimidazo[5,1-d]-1,2,3,5-tetrazin-4-(3H)-one (specific activity 26.3 mCi mmole$^{-1}$) was kindly supplied by Dr John Slack (Aston Molecules Ltd, Birmingham, UK). Cell suspensions ($5 \times 10^6$ ml$^{-1}$) were equilibrated at 4°C and treated with 200 μM of the labelled drug. $10^6$ cells were pipetted into eppendorf tubes and centrifuged through 250 μl of an oil mixture (4:1 'Three-in-One'/Dow Corning silicone oil). The aqueous layer was aspirated and the oil layer gently washed with a further 300 μl of saline. After centrifugation both layers were aspirated, the cell pellet dissolved in Protosol and added to scintillation vials containing Optiphase.

### Results

*Cytotoxicity studies*

The data in Table I and Figure 1 show a reasonable correlation between the sensitivity (as measured by the concentration which gives 50% inhibition of growth or $IC_{50}$) of tumour cell lines to temozolomide ($r = 0.87$) or CCNU ($r = 0.92$) and their ATase content. The slopes are nearly parallel except that CCNU is approximately five times more toxic on a molar basis. One exception was the MCF-7 line which is moderately sensitive to temozolomide and has a relatively high ATase activity. Cell lines pretreated with a non-toxic dose of BG were up to 3.5-fold more and 6-fold more sensitive to temozolomide and CCNU respectively.

**Figure 1** Cytotoxicity ($IC_{50}$) of temozolomide (□) and CCNU (X) versus cellular ATase levels in the human tumour cell lines (in order of increasing ATase levels): ZR-75-1, U87MG, U373, LS174T, LOVO, MCF-7 and MAWI.

**Figure 2** Cytotoxicity of temozolomide in pZipneoSV(X)1-transfected (O,□) or phAT-transfected (Δ,∇) XP-derived cell lines in the presence (□,Δ) or absence (O,∇) of 10 μM BG. Error bars indicate ± 1 s.d.

The control XP cells (transfected with pZipneoSV(X)1 (Fan *et al.*, 1990), which express barely detectable levels of ATase, are 4–5-fold more sensitive to temozolomide or the CCNU-related agent mitozolomide than the human ATase cDNA-transfected cells (Table I). In a colony forming assay for the cytotoxicity of temozolomide (Figure 2), BG pretreatment showed a similar degree of potentiation for the human ATase-transfected XP cells as for the tumour cells, but had no measurable effect on the control XP cells, which do not express ATase. Although BG depleted the ATase activity in the former cells (see below), they remained more resistant to temozolomide than the control pZip transfected fibroblasts.

The repeat dosing schedule showed dramatic potentiation of temozolomide toxicity by BG in MAWI and MCF-7 cells (Figure 3, Table II): after treatment with five 24 h doses the former cell line was over 300-fold more sensitive when BG was present. Multiple doses of temozolomide, by itself, were not more toxic than a single 24 h dose in either cell line. In a similar experiment on U373 cells, which have a low level of ATase, the presence of BG caused only a 3-fold potentiation, after four 24 h doses.

*Alkyltransferase levels*

We found that the concentrations of BG used in this study rapidly reduced to an undetectable level (data not shown), the initially high ATase content of MAWI cells and human ATase cDNA transfected XP fibroblasts. HPLC analysis showed that BG was stable in tissue culture medium for at least 24 h at 37°C.

**Table I** Single dose cytotoxicity

| | Temozolomide | | | CCNU | | | |
|---|---|---|---|---|---|---|---|
| *Cell line* | *$IC_{50}$ [– BG] (μM)* | *$IC_{50}$ [+ BG] (μM)* | *Ratio[a]* | *$IC_{50}$ [– BG] (μM)* | *$IC_{50}$ [+ BG] (μM)* | *Ratio[a]* | *ATase (fmol mg$^{-1}$ protein)* |
| *Breast* | | | | | | | |
| ZR-75-1 | 32 | 23 | 1.4 | 12 | 25 | 0.5 | <10 |
| MCF-7 | 325 | 171 | 1.9 | 70 | 31 | 2.2 | 581.3 |
| *Astrocytoma* | | | | | | | |
| U87MG | 172 | 131 | 1.3 | 28 | 8.8 | 3.2 | 21.9 |
| U373 | 131 | 78 | 1.7 | 15 | 12 | 1.2 | 53.2 |
| *Colorectal* | | | | | | | |
| LS174T | 873 | 632 | 1.4 | 73 | 13 | 5.7 | 199.6 |
| LOVO | 848 | 323 | 2.6 | 92 | 32 | 2.9 | 529.0 |
| MAWI | 1173 | 335 | 3.5 | 133 | 30 | 4.4 | 992.3 |
| *XP lines* | | | | | | | |
| pZip | 23[b] | – | – | (0.8)[b,c] | – | – | <2 |
| phAT | 100[b] | – | – | (4.2)[b,c] | – | – | 1240 |

Cells were exposed to ± o$^6$-benzylguanine (BG) prior to a single dose of temozolomide or CCNU. [a]$IC_{50}$ [– BG]/$IC_{50}$ [+ BG]. [b]Results obtained by MTT assay (Wasserman *et al.*, 1988). [c]Figures in parentheses refer to mitozolomide.

**Table II** Repeated dose temozolomide cytotoxicity ($IC_{50}$)

| *Cell line* | *Day 1* | | *Day 2* | | *Day 3* | | *Day 4* | | *Day 5* | |
|---|---|---|---|---|---|---|---|---|---|---|
| | *− BG* | *+ BG* | *− BG* | *+ BG* | *− BG* | *+ BG* | *− BG* | *+ BG* | *− BG* | *+ BG* |
| U373 | | | | | | | 51 | 18 | | |
| MAWI | 319 | 196 | 350 | 59 | 383 | 21 | 383 | 7.2 | 326 | 1.0 |
| MCF-7 | 319 | 89 | 319 | 51 | 375 | 11 | | | | |

Cells were exposed to ± $O^6$-benzylguanine (BG) prior to repeated daily doses of temozolomide.

We also investigated the temozolomide concentration range, following a 3 h incubation, which caused a decrease in the ATase content of U373, MCF-7, LOVO and MAWI cell lines. There was a 50% reduction at 50–100 μM for each line (Figure 4), despite a 3–4-fold difference in the single dose temozolomide cytotoxicity between MCF-7 and the colorectal lines (LOVO and MAWI). We found a similar reduction in the more sensitive U373 line, although the ATase levels were close to the detection limit of the assay.

To eliminate the possibility of differences in temozolomide transport we studied the cell uptake of the [$^{14}$C]-labelled compound by the most sensitive and resistant cell lines (ZR-75-1 and MAWI respectively). Figure 5 shows that uptake was very rapid at 4°C, being complete within 5 min in both cell lines. Similar amounts of drug were found in both cell lines when adjusted for protein concentration. Rapid uptake at 4°C was consistent with passive diffusion of temozolomide previously shown in two lymphoid lines (Bull & Tisdale, 1987).

### Discussion

The dose-limiting toxicity of chloroethylating agents such as mitozolomide and the chloroethylnitrosoureas is severe myelosuppression, whereas temozolomide is tolerated at approximately ten times the MTD of mitozolomide (Newlands *et al.*, 1985; Newlands *et al.*, 1992). It is reasonable to suggest that this is a function of chloroethylation versus methylation and whilst only the former reaction can lead to DNA crosslinks through initial binding to the $O^6$-position of guanine (Tong *et al.*, 1982), the possibility that other chloroethyl lesions in DNA may be more abundant or more cytotoxic than the methyl equivalents must also be considered (Ludlum, 1990). The observation that ATase can prevent the formation of crosslinks by repairing the precursor $O^6$-chloroethylguanine and that ATase expression can provide protection against cell killing by chloroethylnitrosoureas (Jelinek *et al.*, 1988; Margison *et al.*, 1990; Margison & O'Connor, 1990; Wu *et al.*, 1992) have given rise to attempts to potentiate the cytotoxic effects of chloroethylnitrosoureas using BG in xenografts and this has had some success (Dolan *et al.*, 1990*a*; 1990*b*; 1991). The question of whether normal tissues would be equally affected is only beginning to be addressed (Fairbairn and Margison, submitted).

In the present report, we have shown a parallel toxicity for temozolomide and CCNU (after 1 h drug exposure) with a number of human tumour cell lines, correlating with their ATase content. This suggests that methylation at the $O^6$-position of guanine in DNA is an important cytotoxic lesion for temozolomide. Pretreating cells with BG causes a modest (<4-fold) increase in temozolomide toxicity, presumably because temozolomide itself causes partial depletion of the ATase protein through DNA methylation. The degree of enhancement for temozolomide and CCNU are of a similar order of magnitude. In a colony assay, human ATase cDNA-transfected fibroblasts pretreated with BG remained more resistant to temozolomide than control transfected fibroblasts, although the ATase protein was eliminated. This is unlikely to be due to differences in temozolomide transport and may simply reflect resynthesis of ATase by the phAT fibroblasts to diminish the effect of pretreatment with the inhibitor.

We found a major potentiation by BG of temozolomide toxicity (300-fold) in the MAWI cell line after 5 days treatment. A similar degree of enhancement was seen in MCF-7 cells which also contain high levels of ATase, but only a

**Figure 3** Cytotoxicity ratio of repeated daily doses of temozolomide in MAWI ((X), MCF-7 (∇) or U373 (#) human tumour cell lines of drug only, $IC_{50}$ (−BG), compared to preincubation with BG, $IC_{50}$ (+BG).

**Figure 4** Effect of increasing concentrations of temozolomide on ATase levels in the human tumour cell lines: LOVO (□), MAWI (X), MCF-7 (∇), U373 (#).

**Figure 5** Uptake of radiolabel at 4°C by cells treated with $^{14}$C-temozolomide. MAWI (□), ZR-75-1 (X).

small effect in U373 cells which have low levels. This implies that the continued presence of the ATase inhibitor permits a build up of DNA damage. It is interesting that a flow cytometry study (Catapano *et al.*, 1987) has shown that temozolomide induces a block in S (late)-$G_2$-M both *in vitro* and in mice. This block occurs at least two cell divisions after drug treatment, in contrast to many DNA-interacting agents, including mitozolomide (Broggini *et al.*, 1986), which induce a pre-mitotic block a few hours after drug treatment.

Pharmacokinetic studies (Newlands *et al.*, 1992) have shown that patients receiving temozolomide on a repeated dose schedule attain a maximum plasma concentration of about 50 $\mu$M, which is similar to the $IC_{50}$ values of our cell lines with low levels of ATase. Distribution studies in mice show that temozolomide like mitozolomide (Brindley *et al.*, 1986, unpublished results) has good tissue distribution including penetration into the tumour tissue and across the blood-brain barrier. It is also known that the brain contains low levels of ATase in comparison with other tissues in the body such as liver and spleen (Citron *et al.*, 1991; Pegg & Byers, 1992). Further increases in activity might be obtained by ATase depletion but potentiation of cytotoxicity may also occur in normal cells (Fairbairn & Margison, submitted). It may be that BG or a derivative which is selectively accumulated by tumours, could extend the range of temozolomide-sensitive tumours.

This work was supported by the Cancer Research Campaign.

### References

BRINDLEY, C.J., ANTONIW, P. & NEWLANDS, E.S. (1986). Plasma and tissue distribution of mitozolomide in mice. *Br. J. Cancer*, **53**, 91–97.

BROGGINI, M., ERBA, E., MORASCA, L., HORGAN, C. & D'INCALCI, M. (1986). *In vivo* studies with the novel anticancer agent mitozolomide (NSC 353451) on Lewis lung carcinoma. *Cancer Chemother. Pharmacol.*, **16**, 125–128.

BULL, V.L. & TISDALE, M.J. (1987). Antitumour imidazotetrazines-XVI. Macromolecular alkylation by 3-substituted imidazotetrazinones. *Biochem. Pharmacol.*, **36**, 3215–3220.

BULL, V.L. (1988). Studies on the mode of cytotoxicity of imidazotetrazinones. PhD Thesis, Aston University.

CATAPANO, C.V., BROGGINI, M., ERBA, E., PONTI, M., MARIANI, L., CITTI, L. & D'INCALCI, M. (1987). *In vitro* and *in vivo* methazolastone-induced DNA damage and repair in L-1210 leukemia sensitive and resistant to chloroethylnitrosoureas. *Cancer Res.*, **47**, 4884–4889.

CITRON, M., DECKER, R., CHEN, S., SCHNEIDER, S., GRAVER, M., KLEYNERMAN, L., KAHN, L.B., WHITE, A., SCHOENHAUS, M. & YAROSH, D. (1991). $O^6$-methylguanine-DNA methyltransferase in human normal and tumour tissue from brain, lung and ovary. *Cancer Res.*, **51**, 4131–4134.

DOLAN, M.E., MITCHELL, R.B., MAMMERT. C., MOSCHEL, R.C. & PEGG, A.E. (1991). Effect of $O^6$-benzylguanine analogues on sensitivity of human tumour cells to the cytotoxic effects of alkylating agents. *Cancer Res.*, **51**, 3367–3372.

DOLAN, M.E., MOSCHEL, R.C. & PEGG, A.E. (1990*a*). Depletion of mammalian $O^6$-alkylguanine-DNA alkyltransferase activity by $O^6$-benzylguanine provides a means to evaluate the role of this protein in protection against carcinogenic and therapeutic alkylating agents. *Proc. Natl Acad. Sci. USA*, **87**, 5368–5372.

DOLAN, M.E., STINE, L., MITCHELL, R.B., MOSCHEL, R.C. & PEGG, A.E. (1990*b*). Modulation of mammalian $O^6$-alkylguanine-DNA alkyltransferase *in vivo* by $O^6$benzylguanine and its effect on the sensitivity of a human glioma tumor to 1-(2-chloroethyl)-3-(4-methylcyclohexyl)-1 nitrosourea. *Cancer Commun.*, **2**, 371–377.

FAN, C.-Y., POTTER, P.M., RAFFERTY, J.A., WATSON, A.J., CAWKWELL, L., SEARLE, P.F., O'CONNOR, P.J. & MARGISON, G.P. (1991). Expression of a human $O^6$-alkylguanine-DNA-alkyltransferase cDNA in human cells and transgenic mice. *Nucleic Acids Res.*, **18**, 5723–5727.

FAIRBAIRN, L.J. & MARGISON, G.P. Resistance of human primary bone marrow cells to temozolomide is ablated by $O^6$-benzylguanine. *Br. J. Cancer*. (submitted).

JELINEK, J., KLEIBL, K., DEXTER, T.M. & MARGISON, G.P. (1988). Transfection of murine multi-potent haemopoietic stem cells with an E.coli DNA alkyltransferase gene confers resistance to the toxic effects of alkylating agents. *Carcinogenesis*, **9**, 81–87.

LEE, S.M., THATCHER, N. & MARGISON, G.P. (1991). $O^6$-alkylguanine-DNA alkyltransferase depletion and regeneration in human peripheral lymphocytes following Dacarbazine and Fotemustine. *Cancer Res.*, **51**, 619–623.

LUDLUM, D.B. (1990). DNA Alkylation by the haloethylnitrosoureas: nature of modifications produced and their enzymatic repair or removal. *Mutat. Res.*, **233**, 117–126.

MARGISON, G.P. & O'CONNOR, P.J. (1990). Biological consequences of reactions with DNA: Role of specific lesions. In *Chemical Carcinogenesis and Mutagenesis*. Handbook of Experimental Pharmacology Vol. 94/1, Grover, P.L., & Phillips, D.H. (ed.) pp. 547–571. Springer: Heidelberg.

MARGISON, G.P., HARRIS, L., CERNAKOVA, L., VLCKOVA, V., BROZMANOVA, J., KLEIBL, K. & SKORVAGA, M. (1990). $O^6$-alkylguanine-DNA-alkyltransferase gene expression and the toxicity of triazenes. In *Triazenes: Chemical, Biological and Structural Aspects*, Giraldi, T., Connors, T.A., & Cartei, G. (ed.) pp. 161–172. Plenum Press.

MORTEN, J.E., BAYLEY, L., WATSON, A.J., WARD, T.H., POTTER, P.M., RAFFERTY, J.A. & MARGISON, G.P. (1992). Upregulation of $O^6$-alkylguanine-DNA-alkyltransferase expression and the presence of double minute chromosomes in alkylating agent selected Chinese hamster cells. *Carcinogenesis*, **13**, 483–487.

NEWLANDS, E.S., BLACKLEDGE, G., SLACK, J.A., GODDARD, C., BRINDLEY, C.J., HOLDEN, L. & STEVENS, M.F.G. (1985). Phase I clinical trial of mitozolomide. *Cancer Treat. Rep.*, **69**, 801–805.

NEWLANDS, E.S., BLACKLEDGE, G.R.P., SLACK, J.A., RUSTIN, G.J.S., SMITH, D.B., STUART, N.S.A., QUARTERMAN, C.P., HOFFMAN, R., STEVENS, M.F.G., BRAMPTON, M.H. & GIBSON, A.C. (1992). Phase I trial of temozolomide (CCRG 81045: M&B 39831: NSC 362856). *Br. J. Cancer*, **65**, 287–291.

O'REILLY, S.M., NEWLANDS, E.S., GLASER, M.G., BRAMPTON, M., RICE-EDWARDS, J.M., ILLINGWORTH, R.D., RICHARDS, P.G., KENNARD, C., COLQUHOUN, I.R., LEWIS, P. & STEVENS, M.F.G. Temozolomide: A new oral cytotoxic chemotherapeutic agent with promising activity against primary brain tumours. *Eur. J. Cancer*, (in press).

PEGG, A.E. & BYERS, T.L. (1992). Repair of DNA containing $O^6$-alkylguanine. *FASEB J.*, **6**, 2302–2310.

SKEHAN, P., STORENG, R., SCUDIERO, D., MONKS, A., MCMAHON, J., VISTICA, D., WARREN, J.T., BOKESCH, H., KENNEY, S. & BOYD, M.R. (1990). New Colorimetric Assay for Anti-Cancer Drug Screening. *J. Natl Cancer Inst.*, **82**, 1107–1118.

STEVENS, M.F., HICKMAN, J.A., LANGDON, S.P., CHUBB, D., VICKERS, L., STONE, R., BAIG, G., GODDARD, C., GIBSON, N.W. & SLACK, J.A. (1987). Antitumour activity and pharmacokinetics in mice of 8-carbamoyl-3-methyl-imidazo[5,1-d]-1,2,3,5-tetrazin-4(3H)-one (CCRG 81045; M&B 39831), a novel drug with potential as an alternative to dacarbazine. *Cancer Res.*, **47**, 5846–5852.

STEVENS, M.F.G., HICKMAN, J.A., STONE, R., GIBSON, N.W., BAIG, G.U., LUNT, E. & NEWTON, C.G. (1984). Antitumour imidazotetrazines 1. Synthesis and chemistry of 8-carbamoyl-3-(2-chloroethyl)imidazo[5,1-d]-1,2,3,5-tetrazin-4(3H)-one, a novel broad spectrum antitumour agent. *J. Med. Chem.*, **27**, 196–201.

TANO, K., SHIOTA, S., COLLIER, J., FOOTE, R.S. & MITRA, S. (1990). Isolation and structural characterization of a cDNA clone encoding the human DNA repair protein for $O^6$-alkylguanine. *Proc. Natl Acad. Sci. USA*, **87**, 686–690 & 3253 (erratum).

TONG, W.P., KIRK, M.C. & LUDLUM, D.B. (1982). Formation of the crosslink 1-[$N^3$-deoxycytidyl],2-[$N^1$-deoxyguanosyl]-ethane in DNA treated with N,N'-bis(2-chloroethyl)-N-nitrosourea. *Cancer Res.*, **42**, 3102–3105.

WASSERMAN, T.H. & TWENTYMAN, P. (1988). Use of a colorimetric microtiter (MTT) assay in determining the radiosensitivity of cells from murine solid tumours. *Int. J. Radiat. Oncol. Biol. Phys.*, **15**, 699–702.

WU, Z., CHAN, C.-L., EASTMAN, A. & BRESNICK, E. (1992). Expression of human $O^6$-methylguanine-DNA methyltransferase in a DNA excision repair deficient Chinese hamster ovary cell line and its response to certain alkylating agents. *Cancer Res.*, **52**, 32–35.


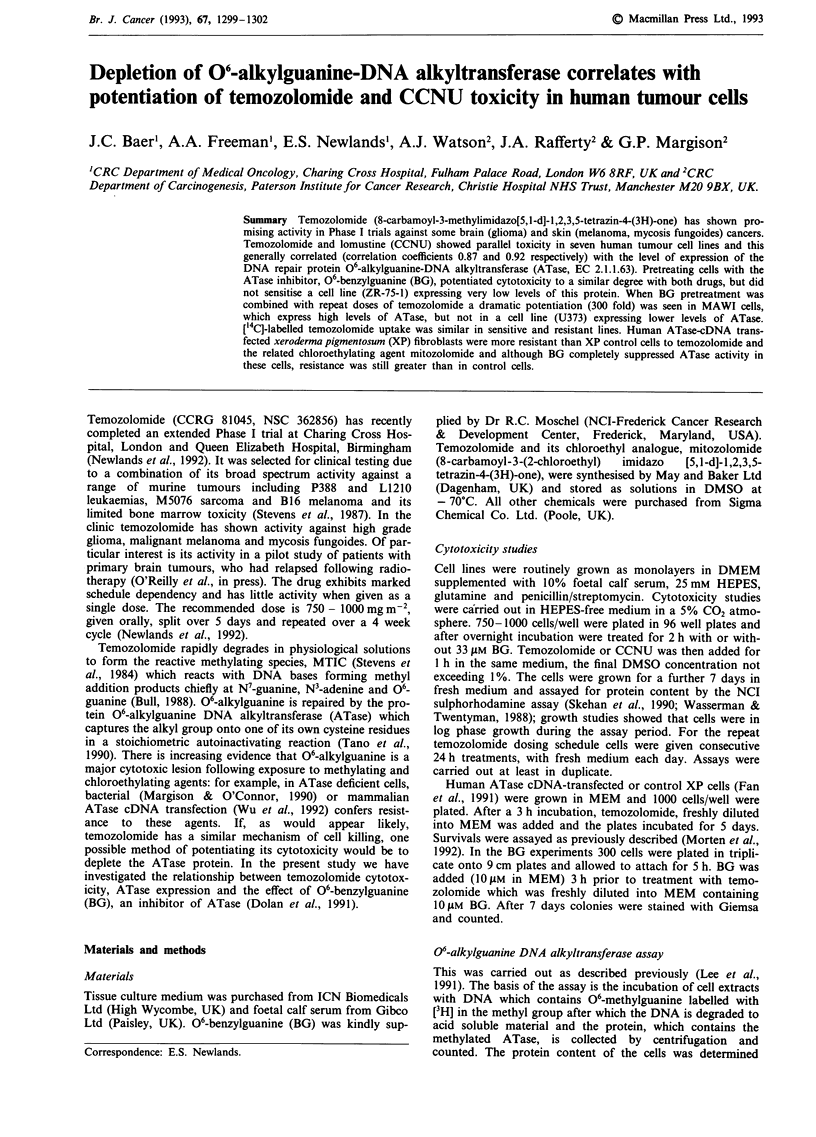

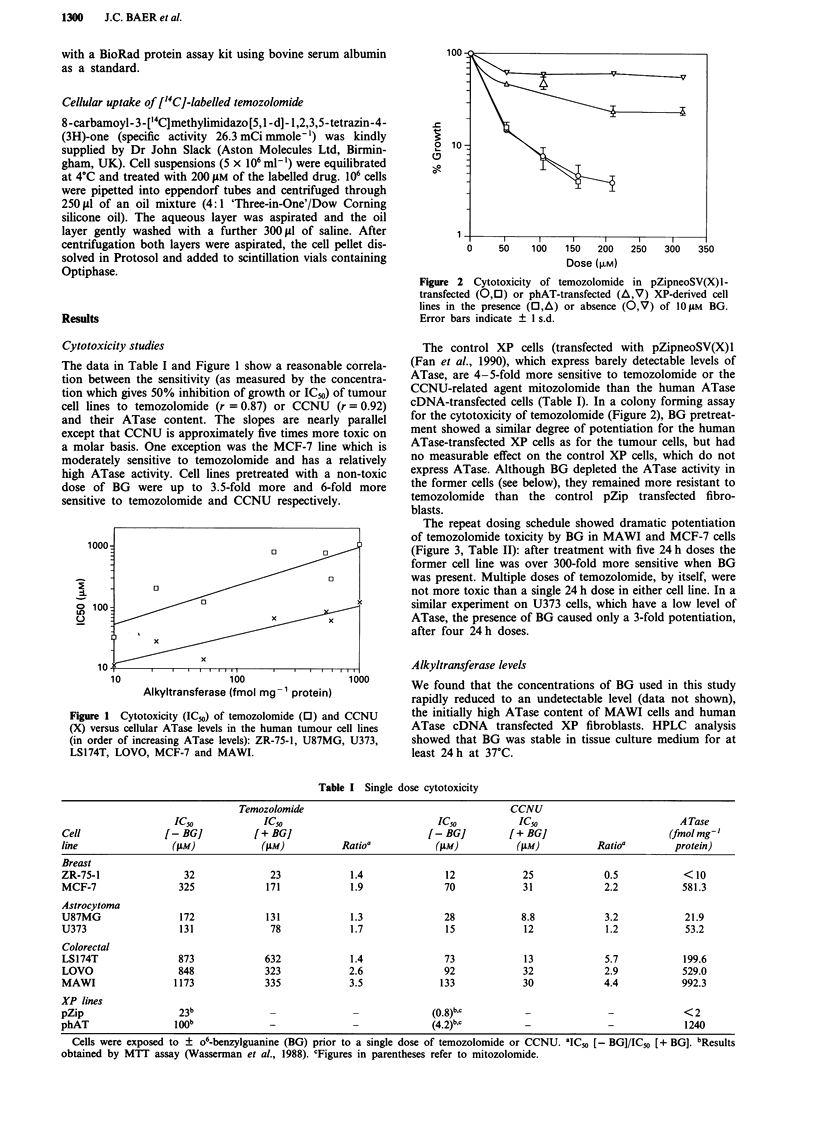

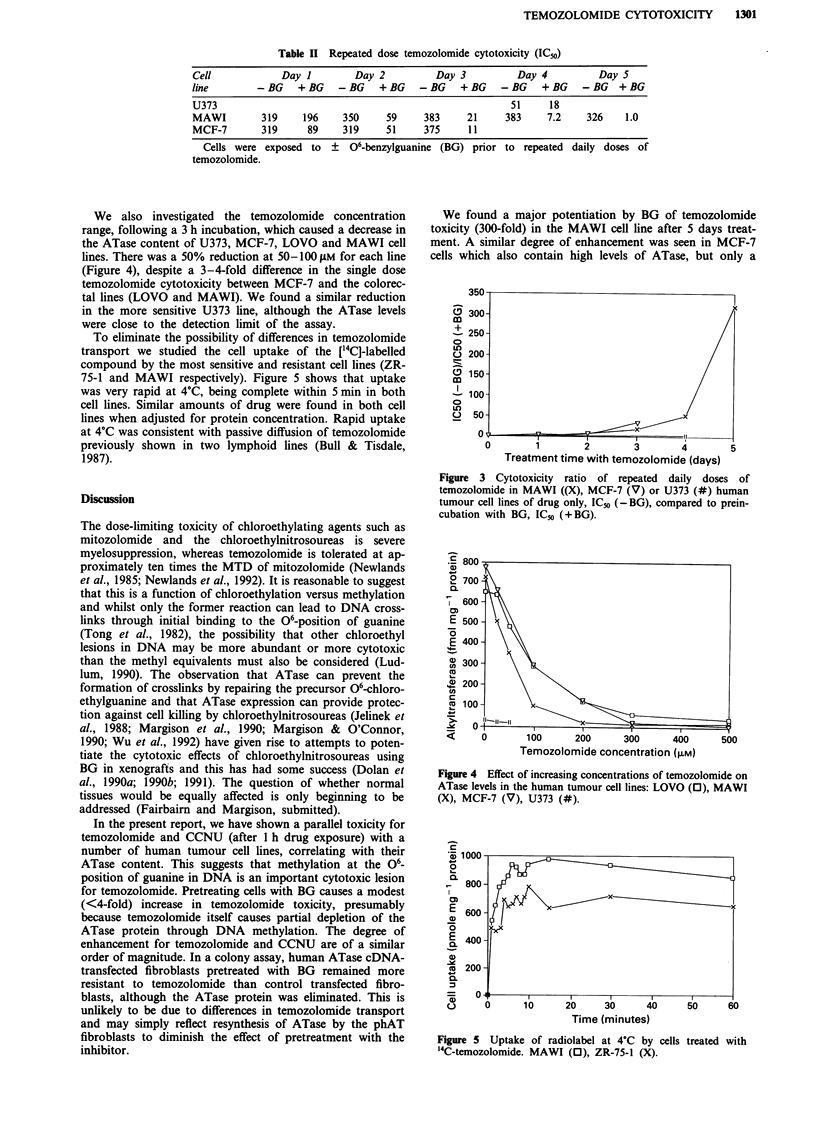

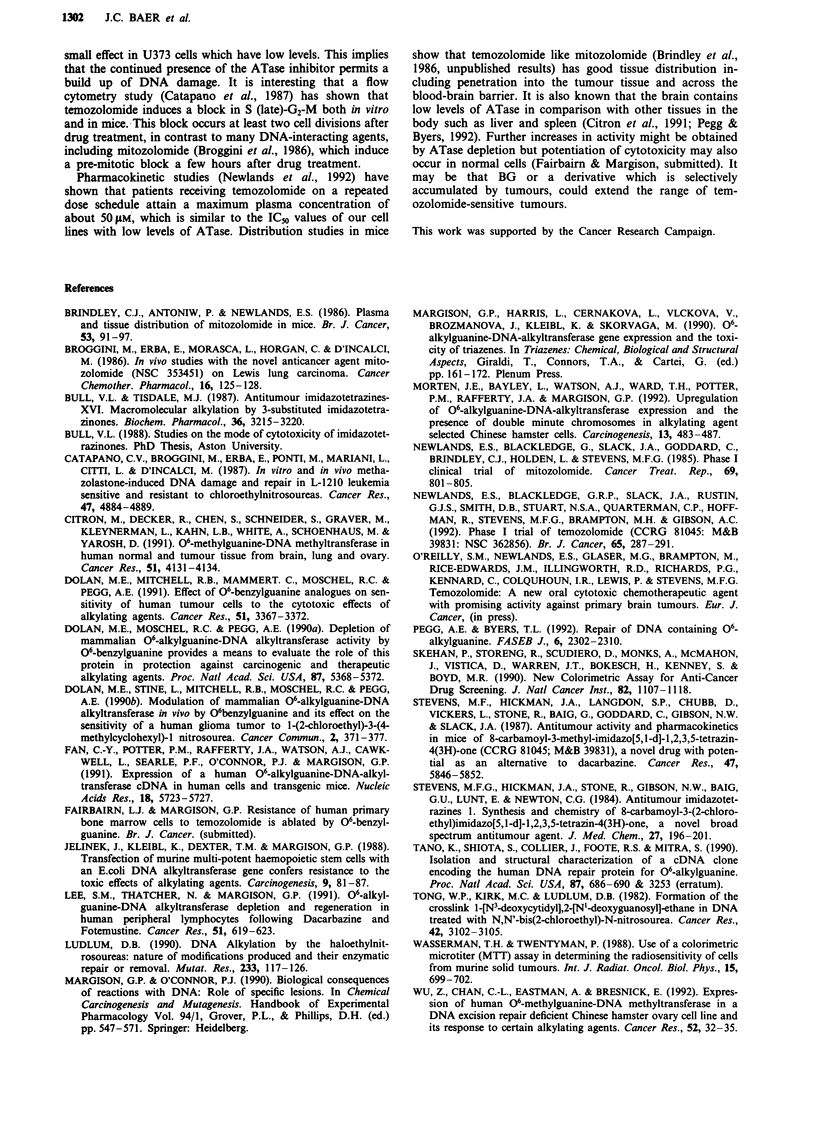


## References

[OCR_00470] Brindley C. J., Antoniw P., Newlands E. S. (1986). Plasma and tissue disposition of mitozolomide in mice.. Br J Cancer.

[OCR_00475] Broggini M., Erba E., Morasca L., Horgan C., D'Incalci M. (1986). In vivo studies with the novel anticancer agent mitozolomide (NSC 353451) on Lewis lung carcinoma.. Cancer Chemother Pharmacol.

[OCR_00481] Bull V. L., Tisdale M. J. (1987). Antitumour imidazotetrazines--XVI. Macromolecular alkylation by 3-substituted imidazotetrazinones.. Biochem Pharmacol.

[OCR_00490] Catapano C. V., Broggini M., Erba E., Ponti M., Mariani L., Citti L., D'Incalci M. (1987). In vitro and in vivo methazolastone-induced DNA damage and repair in L-1210 leukemia sensitive and resistant to chloroethylnitrosoureas.. Cancer Res.

[OCR_00497] Citron M., Decker R., Chen S., Schneider S., Graver M., Kleynerman L., Kahn L. B., White A., Schoenhaus M., Yarosh D. (1991). O6-methylguanine-DNA methyltransferase in human normal and tumor tissue from brain, lung, and ovary.. Cancer Res.

[OCR_00504] Dolan M. E., Mitchell R. B., Mummert C., Moschel R. C., Pegg A. E. (1991). Effect of O6-benzylguanine analogues on sensitivity of human tumor cells to the cytotoxic effects of alkylating agents.. Cancer Res.

[OCR_00510] Dolan M. E., Moschel R. C., Pegg A. E. (1990). Depletion of mammalian O6-alkylguanine-DNA alkyltransferase activity by O6-benzylguanine provides a means to evaluate the role of this protein in protection against carcinogenic and therapeutic alkylating agents.. Proc Natl Acad Sci U S A.

[OCR_00517] Dolan M. E., Stine L., Mitchell R. B., Moschel R. C., Pegg A. E. (1990). Modulation of mammalian O6-alkylguanine-DNA alkyltransferase in vivo by O6-benzylguanine and its effect on the sensitivity of a human glioma tumor to 1-(2-chloroethyl)-3-(4-methylcyclohexyl)-1-nitrosourea.. Cancer Commun.

[OCR_00525] Fan C. Y., Potter P. M., Rafferty J., Watson A. J., Cawkwell L., Searle P. F., O'Connor P. J., Margison G. P. (1990). Expression of a human O6-alkylguanine-DNA-alkyltransferase cDNA in human cells and transgenic mice.. Nucleic Acids Res.

[OCR_00535] Jelinek J., Kleibl K., Dexter T. M., Margison G. P. (1988). Transfection of murine multi-potent haemopoietic stem cells with an E. coli DNA alkyltransferase gene confers resistance to the toxic effects of alkylating agents.. Carcinogenesis.

[OCR_00541] Lee S. M., Thatcher N., Margison G. P. (1991). O6-alkylguanine-DNA alkyltransferase depletion and regeneration in human peripheral lymphocytes following dacarbazine and fotemustine.. Cancer Res.

[OCR_00547] Ludlum D. B. (1990). DNA alkylation by the haloethylnitrosoureas: nature of modifications produced and their enzymatic repair or removal.. Mutat Res.

[OCR_00567] Morten J. E., Bayley L., Watson A. J., Ward T. H., Potter P. M., Rafferty J. A., Margison G. P. (1992). Upregulation of O6-alkylguanine-DNA-alkyltransferase expression and the presence of double minute chromosomes in alkylating agent selected Chinese hamster cells.. Carcinogenesis.

[OCR_00583] Newlands E. S., Blackledge G. R., Slack J. A., Rustin G. J., Smith D. B., Stuart N. S., Quarterman C. P., Hoffman R., Stevens M. F., Brampton M. H. (1992). Phase I trial of temozolomide (CCRG 81045: M&B 39831: NSC 362856).. Br J Cancer.

[OCR_00574] Newlands E. S., Blackledge G., Slack J. A., Goddard C., Brindley C. J., Holden L., Stevens M. F. (1985). Phase I clinical trial of mitozolomide.. Cancer Treat Rep.

[OCR_00595] Pegg A. E., Byers T. L. (1992). Repair of DNA containing O6-alkylguanine.. FASEB J.

[OCR_00599] Skehan P., Storeng R., Scudiero D., Monks A., McMahon J., Vistica D., Warren J. T., Bokesch H., Kenney S., Boyd M. R. (1990). New colorimetric cytotoxicity assay for anticancer-drug screening.. J Natl Cancer Inst.

[OCR_00605] Stevens M. F., Hickman J. A., Langdon S. P., Chubb D., Vickers L., Stone R., Baig G., Goddard C., Gibson N. W., Slack J. A. (1987). Antitumor activity and pharmacokinetics in mice of 8-carbamoyl-3-methyl-imidazo[5,1-d]-1,2,3,5-tetrazin-4(3H)-one (CCRG 81045; M & B 39831), a novel drug with potential as an alternative to dacarbazine.. Cancer Res.

[OCR_00614] Stevens M. F., Hickman J. A., Stone R., Gibson N. W., Baig G. U., Lunt E., Newton C. G. (1984). Antitumor imidazotetrazines. 1. Synthesis and chemistry of 8-carbamoyl-3-(2-chloroethyl)imidazo[5,1-d]-1,2,3,5-tetrazin-4(3 H)-one , a novel broad-spectrum antitumor agent.. J Med Chem.

[OCR_00621] Tano K., Shiota S., Collier J., Foote R. S., Mitra S. (1990). Isolation and structural characterization of a cDNA clone encoding the human DNA repair protein for O6-alkylguanine.. Proc Natl Acad Sci U S A.

[OCR_00627] Tong W. P., Kirk M. C., Ludlum D. B. (1982). Formation of the cross-link 1-[N3-deoxycytidyl),2-[N1-deoxyguanosinyl]ethane in DNA treated with N,N'-bis(2-chloroethyl)-N-nitrosourea.. Cancer Res.

[OCR_00633] Wasserman T. H., Twentyman P. (1988). Use of a colorimetric microtiter (MTT) assay in determining the radiosensitivity of cells from murine solid tumors.. Int J Radiat Oncol Biol Phys.

[OCR_00639] Wu Z. N., Chan C. L., Eastman A., Bresnick E. (1992). Expression of human O6-methylguanine-DNA methyltransferase in a DNA excision repair-deficient Chinese hamster ovary cell line and its response to certain alkylating agents.. Cancer Res.

